# A Unique Case Report of Filicide-Suicide Involving a ‘Perissos’ Suicidal Act

**DOI:** 10.7759/cureus.73182

**Published:** 2024-11-06

**Authors:** Sidharth Salil, James Rajesh Joseph, Janani Adiaman

**Affiliations:** 1 Forensic Medicine and Toxicology, Velammal Medical College Hospital & Research Institute, Madurai, IND

**Keywords:** asphyxiant, carbon monoxide, carboxyhaemoglobin, filicide suicide, perissos

## Abstract

Carbon monoxide is a potent killer and has a high affinity towards hemoglobin. It forms carboxyhemoglobin, which decreases the oxygen supply to all the vital organs. Thus, it causes multi-organ dysfunction on acute exposure, and its most alluring side effect is a gradual, painless, unaware death. So, it is used as one of the preferred agents of suicide nowadays, especially by the learned population. In India, the number of death cases related to inhalation of carbon monoxide is very high, and it is vastly under-reported. This is a case report of a 35-year-old female who was found dead in the bedroom of her house along with her husband and two daughters. Post-mortem examination of all the deceased revealed signs consistent with poisoning due to inhalation of carbon monoxide gas. An Ultraviolet Spectrometric study performed at the laboratory confirmed the presence of carboxyhemoglobin in the blood. Examination of the scene of the crime concerning this case revealed some findings as to how meticulously this suicide cum homicide was planned by the parents. This case turns out to be a case of filicide suicide, where the parents have committed suicide along with the homicide of their children. The peculiar aspect of this case was that more than one method was used to produce the required asphyxiant, which was newly termed the 'Perissos' suicidal act.

## Introduction

A poison is any substance that can cause injury, illness, or death when ingested, inhaled, or absorbed by the body through any route. Poisoning can occur accidentally or intentionally, and the severity of the effects depends on the type of poison, the route of administration, and the amount of poison entering the body. Carbon monoxide (CO), also called 'silent killer', comes under the chemical asphyxiant category, a colorless, odorless, tasteless, and non-irritant gas, lighter than air, and insoluble in water. Carbon monoxide is readily absorbed on inhalation. It has 200-250 times more affinity for hemoglobin than oxygen [1]. It displaces oxygen from its combination with hemoglobin and forms carboxyhemoglobin. Carboxyhaemoglobin (CO-Hb) formation results in reduced arterial oxygen content and causes tissue hypoxia [2]. CO-Hb formation in the blood depends on a wide variety of factors, including the concentration of inspired CO, duration of CO exposure, pulmonary ventilation, exercise, and health status of a person [3]. CO shows a high affinity not only for hemoglobin but also for other heme proteins such as myoglobin and cytochrome C oxidase, thus affecting myocardium and skeletal muscles. Apoptosis is a key factor responsible for cardiotoxicity (heart failure), neurotoxicity (intracellular oxidative stress), and multi-organ dysfunction (increased vascular permeability) on exposure to CO [3]. The most alluring side effect is it causes a gradual, painless, unaware death [4]. Thus, CO is an agent of choice nowadays among the educated population for committing suicide. In India, the number of death cases related to inhalation of carbon monoxide is very high, but it is vastly under-reported [5].

"Filicide-suicide" is a specific form of homicide-suicide referring to a situation when a parent (or parents) purposefully kills one or more of their own children (filicide) and then commits suicide. It involves situations in which suicide and filicide are linked, denoting a highly unpleasant and intricate family dynamic. Common means of commission include exposure to a drug that causes asphyxia, neglect, abandonment, or a destructive act such as stabbing, strangulation, etc. [6]. Filicide-suicide comes under the ambit of dyadic death(s), which means that the initial death(s) could be a murder followed by the murderers taking their own life, or it could be the simultaneous suicide of two or more persons [6].

Here, we describe a unique case of filicide-suicide in which the parents and the two children died due to inhalation of carbon monoxide, and to be more specific, more than one method was used to produce the required asphyxiant. We believe that there are not many cases of dyadic deaths documented in literature where the lethal agent was produced using multiple methods.

## Case presentation

**Figure 1:**
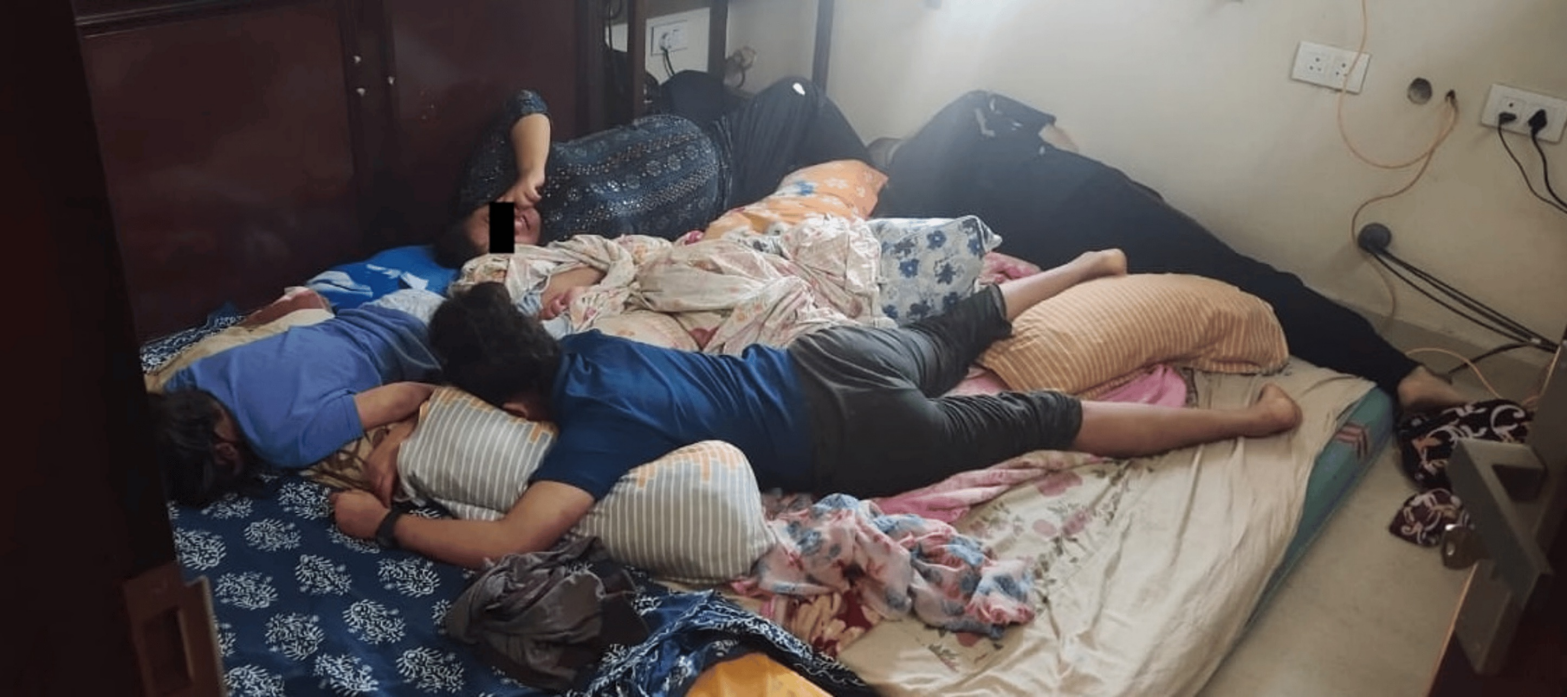
Crime scene photo showing all four family members in their residence bedroom

A 35-year-old female was found unconscious, along with her husband (41 years) and two daughters (8 and 13 years), in the bedroom of their independent residential house (Figure 1). The family was last seen by their maid the previous night. As per records, the room was found to be locked from the inside, with all windows latched and sealed completely. The air conditioner was operating at the time of the crime scene visit. On breaking, the room was hazy at first. After a time of waiting, the team entered the room and found the victims lying on the bed and floor. They also found a cast iron frying pan with small charcoal briquettes. Another pan (smaller in size) containing a white powdery material was also found over the cast iron frying pan. Upon further examination, one packet each of calcium carbonate and zinc was found inside the wall closet of the bedroom. A thorough search of the hallway revealed a printed and signed suicide note, which was carefully tucked in the personal diary of the husband. As per the suicide note, the last paragraph mentioned a warning stating people not to enter the room in haste as they may suffer from the asphyxiating effects of carbon monoxide.

**Figure 2:**
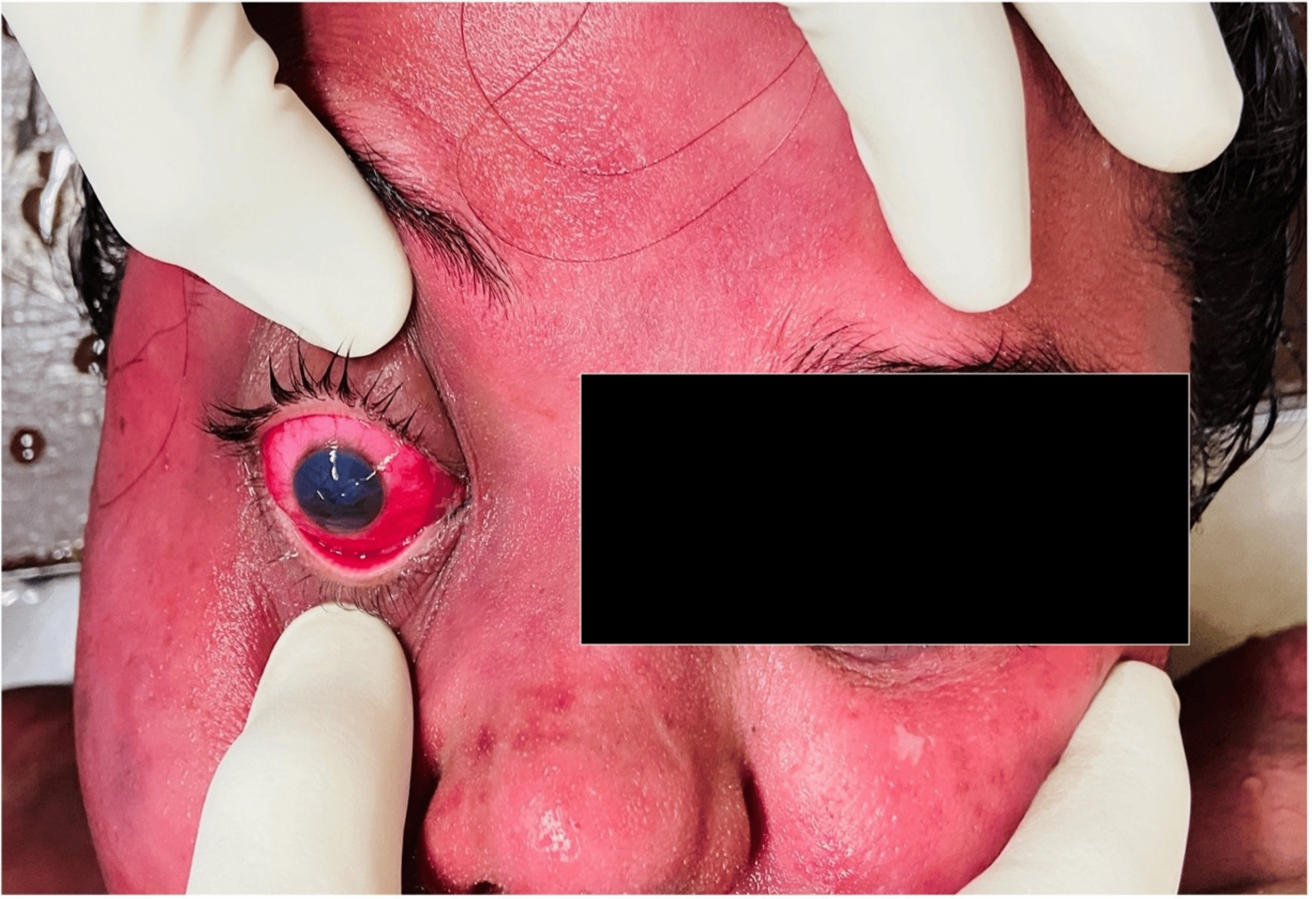
Cherry red discoloration of the skin of the face and conjunctiva

On autopsy examination, the dead body of the woman weighed 100 kg and measured 160 cm in length. Rigor mortis was present all over the body. Cherry red post-mortem lividity was noted on the back and was fixed. Contact pallor was also present. Intense cherry red discoloration with petechial hemorrhages was noted on the conjunctivae, face, and adjoining neck (Figure 2).

**Figure 3:**
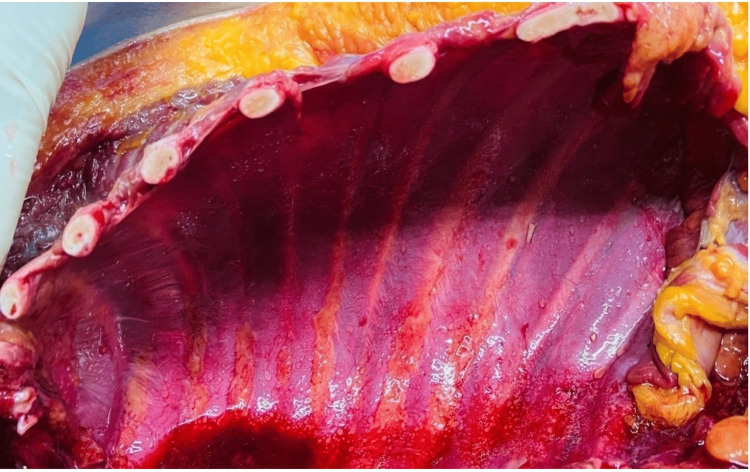
Cherry red discoloration of the intercostal muscles.

Multiple small abrasions, over an area of 6 x 4 cm, were noted on the back of the right elbow. On dissection, the chest and abdominal wall muscles were cherry red (Figure 3).

The lungs were congested and edematous, and their cut sections oozed blood-stained frothy fluid. Duramater and all internal organs showed cherry red discoloration. An ultraviolet spectrometric study of blood showed the presence of carboxyhemoglobin. Also, Hoppe-Seyler's test was positive for carbon monoxide. Further chemical analysis of the samples confirmed the presence of carbon monoxide. Similar autopsy findings were found in the other three dead bodies involved in this case.

## Discussion

Domestic exposure to carbon monoxide and related poisoning is rarely reported in India and remains an unrecognized, rather an underrecognized, problem. In most toxicological studies on the pattern of poisoning cases in India, carbon monoxide poisoning cases are hardly reported [5]. Carbon monoxide poisoning occurs by inhalation of a relatively high concentration of the gas. This is not always accidental; it is also used deliberately as a means of suicide. Cases of homicide by CO poisoning have also been reported [4].

Literature indicates that since the late 1990s, the number of suicides involving carbon monoxide has increased in many countries, especially those in Asia [1]. According to the National Crime Records Bureau (NCRB), 13 CO poisoning cases were reported in 2021 and 10 in 2022 [7].

The predominant sources of CO encountered in poisoning cases are house fires, incomplete combustion of fuels (e.g., charcoal, coal briquette, fuel gas, petroleum) using a burner, heating or cooking equipment with insufficient ventilation or improper maintenance, exhaust gas from vehicles using internal combustion engines and industrial accidents. Carbon monoxide is so fundamentally important that many methods have been developed for its production. A major industrial source of CO is producer gas, formed by the combustion of carbon in the air at high temperatures [8-10]. Here, the initially produced CO2 equilibrates with the remaining hot carbon to give CO (as per Boudouard equilibrium).

Coal when burnt:

In the presence of air, it forms carbon dioxide: C + O_2_ → CO_2_

During an insufficient oxygen supply, carbon monoxide (CO) is formed instead of carbon dioxide: 2C+ O_2_ → 2CO

Since CO is a gas, the reduction process can be driven by heating, exploiting the reaction's positive (favorable) entropy. In the laboratory, CO is formed by heating an intimate mixture of powdered zinc metal and calcium carbonate, which releases CO by the reaction: Zn + CaCO_3_ → ZnO + CaO+ CO.

In this case, carbon monoxide was produced within a secured room, miming a gas chamber-like environment. CO was considered the element of choice for poisoning because of its silent nature, where the victim will slip into unconsciousness within a few minutes of inhalation as it acts on the higher centers of the brain, thereby causing silent death. Investigations revealed that information from suicide-related websites may have facilitated the act.

In this case, it was a well-planned suicide pact (between the parents) coupled with homicide (of the two children), as evident from the suicide note that was recovered from the scene of the crime. The children were the victims, and the parents were the perpetrators. According to published research, most dyadic fatalities take place within families. Nock and Marzuk classify dyadic deaths as familicide-suicide, filicide-suicide, spousal murder-suicide, and extra-familial murder-suicide [11]. Thus, the reported case falls into the subdivision of filicide-suicide.

The term complex suicide means two or more different methods of suicide are applied simultaneously to make sure that the death occurs even if one method fails. In this case, the parents have tried only one method, i.e., death due to asphyxiant inhalation, but they have executed the plan of filicide - suicide in a unique way by using more than one way to ensure that the desired killer agent (asphyxiant) is produced.

## Conclusions

This case of dyadic death does not fall under the ambit of complex suicide. As a result, we have used the term "Perissos", which means "superadded" or "more than what is necessary", to describe this act of suicide. In this suicidal act, the parents have planned to produce the asphyxiant of their choice by more than one method, i.e., one by incomplete combustion of the charcoal briquettes and the second method was executed simultaneously by heating an intimate mixture of powdered zinc metal and calcium carbonate. Through both methods, as mentioned earlier, the parents have produced the needed amount of carbon monoxide, which was used to commit suicide as well as homicide.

## References

[1] Aggrawal A (2016). Textbook of Forensic Medicine and Toxicology. Textbook of Forensic medicine and Toxicology, 1st Ed, New.

[2] Vij K (2013). Textbook of Forensic Medicine & Toxicology: Principles & Practice. https://shop.elsevier.com/books/textbook-of-forensic-medicine-and-toxicology-principles-and-practice/vij/978-81-312-2684-1.

[3] Kinoshita H, Türkan H, Vucinic S, Naqvi S, Bedair R, Rezaee R, Tsatsakis A (2020). Carbon monoxide poisoning. Toxicol Rep.

[4] Ershad M, Melisiotis A, Gaskill Z, Kelly M, Hamilton R (2020). An unusual case of carbon monoxide poisoning from formic and sulfuric acid mixture. Clin Pract Cases Emerg Med.

[5] Sasikumar S, James RJ, Sangeetha R (2022). Profile of poisoning cases in a tertiary care hospital in Tamil Nadu, South India - A 4 year retrospective study. J For Med Sci Law.

[6] Ateriya N, Saraf A, Kanchan T, Meshram VP, Singh Shekhawat R, Setia P (2019). Filicide-suicide: An unusual variant of dyadic death. Med Leg J.

[7] (2024). Accidental deaths and suicides in India year-wise. https://www.ncrb.gov.in/accidental-deaths-suicides-in-india-year-wise.html?year=2022&keyword=.

[8] Suzuki Y, Takeda M, Inamura T (1989). Analyzed gases of burning products collected at fire scenes. Rep Fire Sci Lab.

[9] Kawaraya T, Garivait H (1997). Exhaust gases from new gasoline vehicles in Thailand. Seikatsu Eisei.

[10] Yamanouchi H, Honma N, Shigeno R (1985). A fatal case due to the exhaust of diesel engine. Res Pract Forens Med.

[11] Balcı Y, Ekinci E, Anolay NN (2023). Dyadic death series from an autopsy center: Femicide-suicides cases. Leg Med.

